# Mechanisms involved in hearing disorders of thyroid ontogeny: a literature review

**DOI:** 10.1590/2359-3997000000292

**Published:** 2017-09-04

**Authors:** Caio Leônidas Oliveira de Andrade, Gabriela Carvalho Machado, Luciene da Cruz Fernandes, Jamile Morais de Albuquerque, Luciana Lyra Casais-e-Silva, Helton Estrela Ramos, Crésio de Aragão Dantas Alves

**Affiliations:** 1 Instituto de Ciências da Saúde Universidade Federal da Bahia Salvador BA Brasil Programa de Pós-graduação dos Processos Interativos dos Órgãos e Sistemas (PPgPIOS), Instituto de Ciências da Saúde (ICS), Universidade Federal da Bahia (UFBA), Salvador, BA, Brasil; 2 Departamento de Fonoaudiologia Instituto de Ciências da Saúde Universidade Federal da Bahia Salvador BA Brasil Departamento de Fonoaudiologia, Instituto de Ciências da Saúde, Universidade Federal da Bahia (UFBA), Salvador, BA, Brasil; 3 Departamento de Biorregulação Instituto de Ciências da Saúde UFBA Salvador BA Brasil Laboratório de Neuroimuno-endocrinologia e Toxinologia, Departamento de Biorregulação, Instituto de Ciências da Saúde (ICS), UFBA, Salvador, BA, Brasil; 4 Departamento de Biorregulação Instituto de Ciências da Saúde UFBA Salvador BA Brasil Departamento de Biorregulação, Instituto de Ciências da Saúde (ICS), UFBA, Salvador, BA, Brasil; 5 Faculdade de Medicina Unidade de Endocrinologia Pediátrica Universidade Federal da Bahia Salvador BA Brasil Faculdade de Medicina, Unidade de Endocrinologia Pediátrica, Universidade Federal da Bahia (UFBA), Salvador, BA, Brasil

**Keywords:** Thyroid gland, thyroid diseases, congenital hypothyroidism, hearing disorders, hearing loss

## Abstract

Endocochlear, retrocochlear and/or central origin hearing damage may be related to the absence of appropriate levels of thyroid hormone during morphogenesis and/or auditory system development. Hearing disorders related to the thyroid are not well studied, despite speculation on the pathophysiological mechanisms. The objective of this review was to characterize the main pathophysiological mechanisms of congenital hypothyroidism and to evaluate the relationship with central and peripheral hearing disorders. We conducted a literature review using the databases MedLine, LILACS, Cochrane Library, SciELO, Institute for Scientific Information (ISI), Embase, and Science Direct between July and September on 2016. We identified the studies that address hearing disorder mechanisms on the congenital hypothyroidism. Congenital hypothyroidism may have clinical and subclinical manifestations that affect the auditory system and may be a potential risk factor for hearing impairment. Hearing impairment can severely impact quality-of-life, which emphasizes the importance of monitoring and evaluating hearing during the clinical routine of these patients.

## INTRODUCTION

The development of the auditory system depends on the presence of proper levels of thyroid hormone (TH) (1). Several proteins and the synthesis of multiple enzymes require the normal function of the thyroid gland, and hormones are necessary for the structural formation of the middle and inner ear (2) as well as the central auditory system (3). Therefore, it is possible that congenital hypothyroidism may lead to auditory damage with endocochlear origin, retrocochlear origin and/or central parts of the auditory system (3).

The THs play an important role in the morphogenesis, development and maturation of the auditory pathway. Thus, congenital hypothyroidism (CH) can be a potential risk factor for hearing impairment (HI) (4) if the hormones decrease or are absent during the development of the peripheral and central auditory system structures (5).

While the HI incidence in CH individuals is currently unknown, studies suggest it may affect 20% of carriers (5-7). The rate of hearing disorders in CH patients is approximately 100-fold higher than the euthyroid population and occurs in approximately 1 per 1000 births (6).

Although the CH auditory aspects have been investigated in different experimental models involving both in humans and animals, the pathophysiological mechanisms have not been well explored and are not fully elucidated. This lack of information makes it difficult to comprehend all the processes involved in the possible hearing disorders that this disease may cause.

The aim of this literature review was to evaluate the relationships between CH and both peripheral and central hearing disorders. We focused on the pathophysiological mechanisms involved with these disorders.

## MATERIAL AND METHODS

### Identification and selection of studies

The literature search was conducted using the following electronic databases: MedLine, LILACS, Cochrane Library, SciELO, Institute for Scientific Information (ISI), Embase, and Science Direct. The databases were consulted between July and September 2016. The databases were mined for literature that specifically focused on pathophysiological processes of congenital hypothyroidism and hearing in human and animal models. The following keywords and descriptors were used during the search and were combined in a number of sequences in English, Portuguese and Spanish languages: hypothyroidism, congenital hypothyroidism, thyroid hormone, thyroid gland, thyroid ontogeny versus auditory hearing maturation, cochlear function, middle olivocochlear system, central auditory processing, hearing loss, and hearing test. The selected studies were chosen based on their title and abstract description. The desired outcomes were structural, physiological, and/or biochemical disorders of the auditory system due to impaired function of the thyroid gland. Papers were excluded from the analysis if they addressed hearing disorders in syndromic cases associated with hypothyroidism or other hypothyroidism conditions that were not caused by abnormalities due to the formation or function of the thyroid gland.

## LITERATURE REVIEW

### Congenital hypothyroidism

CH is related to defective TH action due to decreased or absent hormones. CH is the most common metabolic dysfunction in newborn infants. CH affects 1:3000 to 1:4000 births worldwide (8) and 1:2500 births in regions of Brazil (9).

CH etiology is clinically classified as either permanent (80-90%) or transitory (10-20%) (10). The causes of CH are broadly categorized into dyshormonogenesis in 15% of cases and thyroid dysgenesis (TD) in 85% of cases (11-14). Dyshormonogenesis is caused by autosomal recessive mutations of key molecules regulating thyroid hormone synthesis, and thyroid hormone production fails in a structurally sound thyroid gland (15). Conversely, TD is caused by a wide range of different structural malformations in the thyroid that result in a wide variety of different CH phenotypes (16-18). TD is subcategorized into the following classes: 1) thyroid agenesis, which is the most severe form and has a complete lack of thyroid tissue (i.e., both lobes); 2) thyroid hemiagenes, which has one of the thyroid lobes completely missing; 3) thyroid hypoplasia, which is characterized as a smaller gland in the normal position; and 4) thyroid ectopia, which involves an abnormal positioning but the gland rests along the migratory path of the primordium. It is known that 5% of thyroid dysgenesis cases are associated with mutations of the genes responsible for the development of the thyroid follicular cells (e.g., *NKX2.1*, *FOXE1*, *PAX8*, and *TSHR*) and display a complex pathogeny (18,19).

Untreated CH can result in a profound impairment of the somatic growth and central nervous system functional differentiation because THs are essential for metabolic development, growth, and homeostasis.

### Endocochlear mechanisms of congenital hypothyroidism

**Figure 1:**
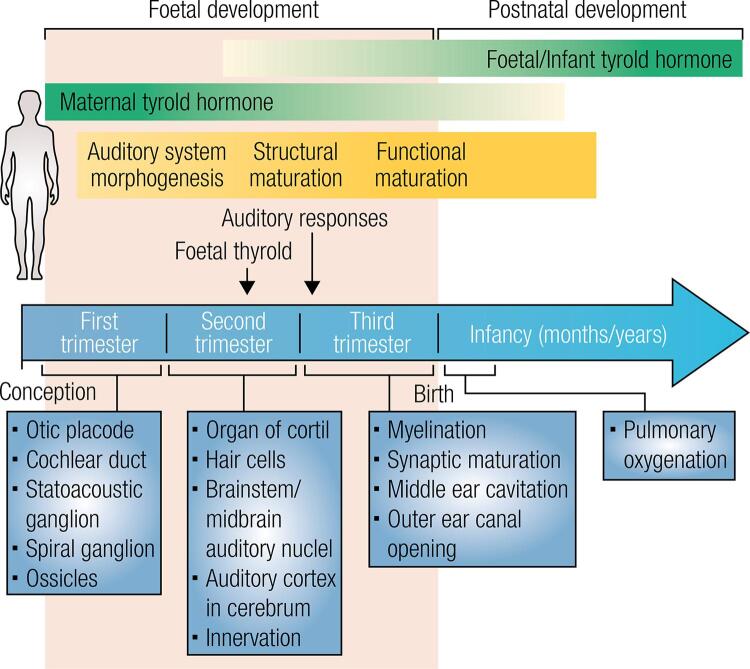
The role of T3 in human auditory system formation and development. In the foetal period, the primary auditory responses and the hearing sensitivity progressively matures until early childhood. During the first quarter, the embryo depends totally on the mother’s thyroid hormones, which are produced in small amounts during the second half of gestation. After birth occurs, there is an increase in the T4 and T3 levels in the newborn. Adapted from Ng and cols., 2013 (23).

Animal model studies demonstrated that thyroxin (T_4_) plays an important role in the development of embryonic inner ear. In CH cases with maturation of the sensory epithelium, the inner ear is injured, which suggests there are periods of sensitivity to THs in the developing cochlea (20). In humans the critical time for hearing maturation corresponds approximately to the gap between the embryonic period and the first year of postnatal life (21) (Figure 1).

The cellular function of THs, specifically the active form triiodothyronine (T_3_), is mediated by the thyroid hormone receptor (TR). TR is a binding transcription factor that changes target gene expression (22). The action of T_3_ on the cochlear sensory cells is partially caused by differential expression of the TH receptor isoforms receptors present in the developing cochlea: α (*THRA*) and β (*THRB*) (23). The expression pattern suggests the cochlea is a direct site of action for THs, which can explain several findings of morphological abnormalities on the spiral organ in hypothyroid rodents (20,24,25).

A delay of THs supply before hearing function development starts results in permanent defects on the cochlea. The deficits of THs can also lead to permanent decreases of the β-tectorin protein levels in the tectorial membrane, which is associated with tectorial membrane structural abnormalities and cochlear function (26).

The outer hair cells (OHC) are highly sensitive to THs serum levels (26). In cases with low hormone levels in the beginning of hearing function, the OHC are poorly differentiated from the other cells in the cochlea. This reduces the number of organelles in the cytoplasm, including ribosomes, endoplasmic reticulum, and mitochondria (27). It is also possible to verify an insufficient formation and changes in microtubule stability with the rise of filamentous actin expression, which increases the stiffness and decreases the cell membrane mass. These changes directly affect the cochlear amplification process (28).

The patients with hypothyroidism show reduced *SLC26A4* gene expression*.* This gene encodes the prestin protein that functions as the motor of the OHC and regulates the cochlear amplification process (29). The reduction in prestin and decreased amplification decrease its distribution in the OHCs membrane (30). Additionally, the K^+^ channel encoded by *KCNQ4* is responsible for endolymphatic potential formation and is also significantly decreased in these conditions (31).

Cumulatively, these factors together with an insufficient opening of the cochlea fluid spaces (inner spiral sulcus, tunnel of Corti, and Nuel’s space) affect the development of cochlear micromechanics (32) and damage both the passive and active cochlea mechanisms (33).

There are also descriptions of abnormalities in numerous afferent dendrites and growth delays of the efferent terminals under the OHCs (34). These findings confirm the hypothesis that the absence or decrease of THs can cause harmful effects to the peripheral auditory system and cochlear function.

### Retrocochlear/central mechanisms on congenital hypothyroidism

Previous studies conducted in animal models focused on central nervous system (CNS) development and how the decrease or absence of THs leads to clinical signs suggesting stagnation of normal CNS maturation in CH cases (34). These findings show an abnormality on the myelination process and subtraction of the axonal projections of the anterior commissure and corpus callosum (35). The abnormalities decrease the pyramidal neurons and cause irregular localization of the corpus callosum neurons. Additionally, there are reduced numbers of microtubules in the neural cytoplasm, changes to the distribution of apical dendrites of the pyramidal neurons (36), and a delay in the cholinergic axons arrival to the hippocampus (37).

Prior studies of the superior auditory pathway have shown reduced levels of the metabolic activity marker deoxyglucose in the following regions: the cochlear nucleus, superior olivary complex, lateral lemniscus nucleus, inferior colliculus, medial geniculate body, and auditory cortex. These data suggest the entire auditory pathway is sensitive to insufficient TH serum levels (38).

A possible explanation for these findings may be associated with the reduced expression of the type 2 deiodinase enzymes, which convert the T_4_ into T_3_ hormone in individuals affected by hypothyroidism and reduce the amount of T_3_ for the auditory centres (39).

Studies of the regions located closer to the spiral organ show changes on the spiral ganglion that cause smaller neurons than found in euthyroid people (40).

The morphology of neurons from the medial olivocochlear tract is altered in CH cases. However, there are no changes to the neuron population and distribution of this tract. If the neurons do not make proper synaptic contact with the OHC (34), then they can contact other cochlear structures (27,32).

Recent evidence indicates the medial olivocochlear tract innervation is more severely affected in the cases with hypofunctional thyroid glands because it remains at an immature stage compared to lateral olivocochlear tract innervation (34).

### Audiological findings of congenital hypothyroidism

CH causes heterogeneity in the disturbances of the auditory structures. Thus, several audiological findings are possible. However, the audiometric disturbances are frequently described as having the following features: sensorineural, bilateral, symmetric, mainly in high frequencies, in a varying degree, and are often mild to moderate severity (6,7,41-46).

Conductive hearing loss and tympanometric abnormalities in addition to acoustic middle ear reflex have also been described in several studies (5,6,7,41-43). However, these conditions are found less frequently and are restricted to cases that are linked to any syndrome.

Once the TH is essential for auditory nervous system neuromaturation, there is evidence indicating a relationship between the presence of symptoms and central auditory processing disorders in CH cases (47).

The electroacoustic tests, such as otoacoustics emissions (OAE), responsible for high frequency sensibility and selectivity show varied results. Therefore, it is possible to see an expressive abnormality of the OAE (43), signal amplitude reduction (44), and an increase in the number of ears classified by the equipment as “fail” due to pre-clinical cochlear susceptibility (48).

The tests used to accurately investigate the neurophysiology of the auditory pathway in CH include the brainstem auditory evoked potentials (BAEP) analysis. The test results show diverse findings, such as prolongation of absolute latency of the I (42,49), III and V (50) waves and increased interpeak interval latency for I-III (50), I-IV (49), and I-V (42). These results suggest there are several alteration sites.

## CONCLUSION

In considering the reviewed content, it has been shown that hypothyroidism, especially in its congenital form, is a potential risk factor for hearing impairment. It can affect hearing from the peripheral structures to central areas that may also lead to inappropriate auditory development. These defects can affect the comprehension and acquisition of acoustic information. Inappropriate auditory development can lead to scholarly, cognitive, language, behavioural and/or social emotional problems. Therefore, it is critical to monitorand evaluate hearing as part of the clinical routine of these patients.
